# Supplementary material for: Transcriptome Analysis Reveals Vimentin-Induced Disruption of Cell–Cell Associations Augments Breast Cancer Cell Migration
Source: Cells. 2022 Dec 13;11(24):4035. doi: 10.3390/cells11244035 (PMC9776984; doi:10.3390/cells11244035)
Supplement: Supplementary file 1 [file cells-11-04035-s001.zip › DEGs and enrichment analysis.zip!/DEGs and enrichment analysis/WT_VIMvsVector KEGG/ALL/WT_VIMvsVector_ALL.pathway_map.zip!/WT_VIMvsVector_ALL.pathway_map/src/hsa05206.html]

hsa05206


- hsa:993

- Up regulated genes

ENSG00000164045(1.78085789611929)

- hsa:1789

- Up regulated genes

ENSG00000088305(1.52753633758326)

- hsa:7042

- Down regulated genes

ENSG00000092969(-2.32734226266555)

- hsa:596

- Up regulated genes

ENSG00000171791(2.03324203294485)

- hsa:5268

- Down regulated genes

ENSG00000206075(-2.52349843817487)

- hsa:4325

- Up regulated genes

ENSG00000156103(1.76289078067105)

- hsa:1869

- Up regulated genes

ENSG00000101412(1.977544660594)

- hsa:1869

- Up regulated genes

ENSG00000101412(1.977544660594)

- hsa:1870

- Up regulated genes

novel.507(3.55620908288126)

- hsa:7431

- Up regulated genes

ENSG00000026025(10.7915998717114)

- hsa:4082

- Down regulated genes

ENSG00000277443(-2.15104303888995)

- hsa:596

- Up regulated genes

ENSG00000171791(2.03324203294485)

- hsa:113130

- Up regulated genes

ENSG00000146670(1.70045445554211)

- hsa:1869

- Up regulated genes

ENSG00000101412(1.977544660594)

- hsa:5268

- Down regulated genes

ENSG00000206075(-2.52349843817487)

Close
